# Strengthening the military stoic tradition: enhancing resilience in military service members and public safety personnel through functional disconnection and reconnection

**DOI:** 10.3389/fpsyg.2024.1379244

**Published:** 2024-09-23

**Authors:** Megan McElheran, Franklin C. Annis, Hanna A. Duffy, Tessa Chomistek

**Affiliations:** ^1^Wayfound Mental Health, Calgary, AB, Canada; ^2^Independent Researcher, Arlington, VA, United States; ^3^Werklund School of Education, University of Calgary, Calgary, AB, Canada

**Keywords:** functional disconnection, functional reconnection, military service members, public safety personnel, first responders, mental health, operational stress injuries, stoicism

## Abstract

This paper addresses operational stress injuries (OSIs) among military service members (SM) and public safety personnel (PSP) resulting from prolonged exposure to potentially psychologically traumatic events (PPTEs). While psychotherapeutic interventions for post-traumatic stress injuries (PTSIs) are well established, there is a significant gap in evidence-based mental health training programs addressing proactive mitigation of negative outcomes from PPTEs. Building on the Functional Disconnection/Functional Reconnection (FD/FR) model, we introduce FD/FR 2, emphasizing early identification and management of psychological risks. FD/FR 2 discusses the practice of emotional suppression, or “pseudo-stoicism,” and its potential negative impact on mental health. By integrating authentic Stoic principles, FD/FR 2 offers practical exercises to enhance resilience and well-being, addressing a critical need in current training approaches for military SM and PSP.

## 1 Introduction

Operational stress injuries (OSIs) frequently manifest in military service members (SM) and public safety personnel (PSP), arising from persistent exposure to potentially psychologically traumatic events (PPTEs; Carleton et al., 2019). The PPTEs experienced by military SM and PSP are associated with higher risk of compromised mental well-being, including various post-traumatic stress injuries (PTSIs; Carleton et al., 2018; Inoue et al., 2021). Encouragingly, the effectiveness of individual psychotherapeutic interventions for PPTE and PTSIs are well established for military SM (Straud et al., 2019) and PSP (Bahji et al., 2022). While these PPTE and PTSIs interventions can serve to support the mental health of military SM and PSP, there are very few evidence-based mental health training programs specifically designed to proactively mitigate the negative mental health outcomes associated with PPTEs (Anderson et al., 2020; Di Nota et al., 2021).

Given the high psychological risks faced by military SM and PSP, we believe that programming should orient and train these communities to identify and address these risks at the beginning of their careers. A review of existing literature found that many military SM and PSP training programs in North America focus on exposing recruits to scenarios that mimic high-stress environments experienced in the workplace (Atkins and Norris, 2012); however, these current training programs often leave members feeling insufficiently prepared to deal with work-related mental health problems (Lentz et al., 2022).

Evidence evaluating the effectiveness of upstream mental health training is building. The “Road to Mental Readiness” is one program that has been provided to SM and PSP since 2006. This program focuses on the provision of psychoeducation and skill building for participants and has demonstrated some effectiveness in reducing stigma for SM and PSP participants (Carleton et al., 2018). Findings from the Before Operational Stress program (which will be described in greater detail in a later section) was developed to address a gap in early career training for SM and PSP. It focuses on education regarding Stoicism and pseudo-stoicism, with the objective of operationalizing mental health skills participants can use to enhance resiliency in active ways throughout their careers (Stelnicki et al., 2021; Ioachim et al., 2024).

McElheran and Stelnicki (2021) assert that military SM and PSP are often encouraged to “harden” their reactions in stressful situations, engaging in pseudo-stoicism (Bassue, 2023). The influence of pseudo-stoicism may lead to over-reliance on ineffective emotional coping mechanisms detrimental to mental health (McElheran and Stelnicki, 2021). In response, they proposed the Functional Disconnection/Functional Reconnection (FD/FR) Model, integrating Stoic philosophy to emphasize functional reconnection during transitions from work to personal contexts. Incorporating Stoicism into intervention programs offers a promising shift toward training approaches that acknowledge the challenges in military and PSP occupations, providing effective coping strategies early in a career. Unlike other resiliency approaches, Stoic philosophy is deeply tied to the Western profession of arms, with many Stoic-influenced military works, such as Marcus Aurelius’s *the Meditations,* supporting self-directed learning and motivation to use Stoic practices among the military community (Annis, 2023).

This paper presents an updated FD/FR model that incorporates Stoic principles into a series of exercises for military SM and PSP to proactively enhance mental resilience and well-being. Each aspect of Stoic philosophy, as interpreted through the FD/FR model, provides valuable insights for the unique challenges faced by military SM and PSP in both professional and personal contexts. Additionally, the application of the FD/FR model within an evidence-informed resiliency-based training program, Before Operational Stress [BOS], will be examined.

## 2 Stoic philosophy

Stoic philosophy resonates with the challenges faced by contemporary military SM and PSP, emphasizing the transformative power of adversity over material wealth or societal status (Sellars, 2006). This alignment is evident when comparing ancient Stoic teachings with modern conceptualizations of posttraumatic growth, often cited by military SM and PSP exposed to PPTEs (Tedeschi et al., 1998). However, we argue that the over-reliance on emotional coping mechanisms, such as suppression and minimization, reflects a misunderstanding of Stoic principles.

In 2021, McElheran and Stelnicki (2021) introduced the Functional Disconnection and Functional Reconnection (FD/FR) model to improve mental health outcomes for military SM and PSP, drawing from Stoic philosophy. They argue that the current application of Stoicism in military and public safety organizations, which emphasizes emotional suppression, deviates from the original intentions of ancient Stoic philosophers. The following section will outline key features of ancient Stoicism and explore the rise of pseudo-stoicism in military and PSP contexts.

### 2.1 Ancient Stoicism

There is no philosophy more deeply connected to the Western military and public safety tradition than Stoicism. Its adoption within PSP cultures is logical (given the paramilitary structures to which PSP sectors adhere). The ancient Stoics collected battlefield-proven approaches to hardening the body and mind from Spartan culture and military veterans (Annis, 2023). Stoicism held virtue as the paramount good. It was the goal in life to live according to reason, seen as a connection to the divine, in harmony with nature. The key features of Stoicism include *the Dichotomy of Control*, *Amori Fati* and *Avoidance of Luxuries* (Sellars, 2006; Sherman, 2007).

#### 2.1.1 Dichotomy of Control

*Dichotomy of Control* focuses individuals on the things in life that exist within their control. Stoics become focused on controlling their judgments, thoughts, and actions while learning to disregard outside factors, including wealth and social positions (Sellars, 2006). Stoics recognized their first impressions and related emotional responses were likely to be wrong. Reflection was required to determine if the correct opinion was held (Enfield, 1792). However, this practice is more of an attitude than a strict direction, as Stoic reflection did not call for the evaluation of every variable within a given scenario. Additionally, the practice of dichotomy of control requires deliberate reflection and would not be possible in emergency situations where cognitive resources are consumed on tasks related to survival, but it could occur when individuals return to situations of relative safety.

#### 2.1.2 Amori Fati

The concept of *Amori Fati* instructs Stoics to “love their fate” and adopt a worldview that interprets even terrible events and hardships in an accepting light. Burdens in life, such as loss or suffering, within this worldview, are ultimately needed and are a means of evolving personal growth and ultimate personal excellence (Sherman, 2007). Both the *Dichotomy of Control* and *Amori Fati* help instill optimism and self-reliance in military and public service contexts.

#### 2.1.3 Avoidance of luxuries

The Stoics were indifferent toward material possessions, instructing individuals to keep their desires small to live well (Sellars, 2006; Sherman, 2007). The desire to live simply offers significant benefits for military SM and PSP, including the reduction of logistical demands and the preservation of available energy reserves (calories) during occupational operations. The lighter a member can learn to live, the quicker they can maneuver on the battlefield and in response to emergency situations while maintaining their ability to think and engage in moral judgment.

### 2.2 Rise of pseudo-stoicism

Pseudo-stoicism, in contrast to authentic Stoic philosophy, leads individuals to actively suppress their emotions (Bassue, 2023). Pseudo-stoicism contends that actions such as crying and other displays of emotion or empathy are commonly deemed as “inappropriate” signals of weakness, fragility, or even incompetence (Bassue, 2023). We contend that the emergence of pseudo-stoicism in the military and public service sectors is likely attributed to changes in education. Recruits and trainees, lacking formal instruction in Stoic philosophy, may seek to emulate the emotional composure of veterans by suppressing their emotions. This effort to reject natural emotions can result in psychological injury and the development of toxic leadership traits. Within the Functional Disconnection/Functional Reconnection framework, we posit that the adoption of pseudo-stoic behaviors is implicitly linked to the prevalence of operational stress injuries among military SM and PSP.

## 3 Functional disconnection/functional reconnection re-visited

In 2021, McElheran and Stelnicki (2021) introduced the Functional Disconnection and Functional Reconnection (FD/FR) model as an alternative to pseudo-stoicism. This framework builds on Whitehead’s (2012) concept of “functional disconnect,” initially applied to physicians delivering terminal diagnoses. McElheran and Stelnicki (2021) noted that similar coping strategies are used by military SM and PSP during critical incidents, requiring emotional and personal detachment to perform their duties effectively.

McElheran and Stelnicki (2021) extended Whitehead’s framework to include “functional reconnection,” addressing the need for military SM and PSP to re-engage with personal experiences post-shift. This reconnection involves reflecting on the impact of exposure to PPTE’s and employing active strategies for processing these experiences. The FD/FR model thus facilitates the transition between occupational and personal contexts, promoting positive coping strategies across various life domains. The model recognizes the necessity of disconnection for professional duties while emphasizing the importance of reconnection for personal well-being (McElheran and Stelnicki, 2021).

## 4 Discussion

### 4.1 FD/FR 2: an updated practical model

Recognizing the need for emotional distance in the work of military SM and PSP, the original FD/FR model emphasizes the importance of functional reconnection for experiential processing. However, the original framework lacked specific application steps. Therefore, we propose an updated FD/FR model that integrates Stoic principles more comprehensively and includes practical exercises to proactively improve mental resilience and well-being. This updated model interprets Stoic philosophy to offer valuable insights tailored to the unique challenges encountered by military SM and PSP in both professional and personal contexts.

Acknowledging that personal excellence is achieved through exposure to loss and suffering, Stoic philosophy is ideal for contemporary military SM and PSP. Rather than pursuing wealth and status, Stoics embraced virtue and recognized that adversity drives personal excellence. This aligns with modern conceptualizations of posttraumatic growth (Tedeschi et al., 1998), often cited by military SM and PSP in understanding chronic PPTE exposure. We propose aligning posttraumatic growth principles (e.g., Tedeschi and Moore, 2016) with FD/FR strategies to challenge pseudo-stoicism and promote true Stoic practices in military and public safety cultures in the following practical ways:

#### 4.1.1 Take the view from above

This Stoic exercise is suitable for both functional disconnection and reconnection. The Stoics advise mindfulness of the transitory nature and recognizing our part in any moment as fleeting. For disconnection, SM and PSP should reflect on their degree of responsibility, particularly in war or critical incidents. It may be that by the time it comes to individual deployment of duty, the outcome of any given situation may already be determined. By practicing the ability to take the view from above, individual distress may be dampened by recognizing the small part any one person has to play in a given situation. For reconnection, Stoics remind us that life is short, encouraging us to focus on activities that align with our values. Practically, this means SM and PSP should actively determine their values and routinely reflect on whether they are living in accordance with these values, particularly in personal relationships and circumstances.

#### 4.1.2 Voluntary discomfort

The Stoics guided how voluntary engagement in difficult or uncomfortable situations could serve to prepare us for future adversities. In modern psychotherapeutic terms, voluntary discomfort can be equated to exposure paradigms used in addressing phobic anxiety (Maund et al., 2019). Such exercises expose oneself to potentially distressing circumstances to enhance tolerance and new learning. In the FD/FR model, we propose engagement in voluntary discomfort practices as a way of life, recognizing that increased tolerance for discomfort can be valuable to the military SM and PSP in both occupational and personal contexts. The physical hardships often faced by these communities during their occupational duties (e.g., long periods without rest, exposure to the elements), and difficult emotional or relational challenges at home (e.g., rebellious children, arguments with a spouse) can benefit from regular practice of voluntary discomfort.

Similarly, we recommend the routine practice of saying “no,” to indulgences to enhance gratitude for what one already has. By intentionally denying cravings, individuals can increase their awareness and appreciation of existing comforts and resources. Existing literature strongly correlates gratitude with happiness (Witvliet et al., 2019), so we advise military SM and PSP to practice this strategy in both occupational and home environments to foster a deeper connection with their current resources.

#### 4.1.3 Focus on what you can control

Military SM and PSP tend to assume undue responsibility for circumstances beyond their control, believing they could have altered adverse outcomes. This misplaced responsibility can negatively impact the outlook and psychological health of military SM and PSP, often because the assumed responsibility is objectively inaccurate. The FD/FR model encourages military SM and PSP to actively assess what variables are within their control, whether on the battlefield, at critical incidents, within their organizations, or in personal contexts. Practicing this assessment diligently can help military SM and PSP more accurately evaluate events and their roles in the outcomes.

#### 4.1.4 Negative visualization

The ancient Stoics engaged in a negative visualization practice known as *futuorum malorum praemediation*. We understand that military SMs and PSP are often familiar with anticipating worst-case scenarios, as this is often required in battlefield and critical incident scenarios. From the perspective of mental wellness, a regular practice of visualizing the worst potential outcome of a scenario (e.g., an argument with a spouse leading to the dissolution of marriage) may enhance these communities’ recognition of the agency they have at their disposal to interrupt the worst-case outcome from coming to fruition. We posit in the FD/FR model that when military SM and PSP believe they can adopt an active approach to coping, their mental health will likely improve.

#### 4.1.5 Summary

In summation, the updated FD/FR model highlights the compatibility of Stoic philosophy with the challenges faced by contemporary military SM and PSP in dealing with potentially traumatic events. The updated model emphasizes that personal excellence, according to Stoicism, arises from exposure to loss and suffering rather than the pursuit of wealth and status. The recommended strategies include taking a broader perspective on one’s role in situations, embracing voluntary discomfort for personal growth, focusing on what can be controlled, practicing routine self-denial for enhanced gratitude, and employing negative visualization to anticipate and interrupt potential negative outcomes.

## 5 Incorporation of the FD/FR model into the before operational stress (BOS) program

The BOS program, developed by Canadian mental health experts, addresses the lack of adequate mental health training for military SM and PSP in North America (Stelnicki et al., 2021). BOS aims to proactively strengthen psychological resilience and provide evidence-informed coping strategies for managing operational stressors. Since 2018, BOS has reached over 70,000 Canadian’s in high-risk professions, including frontline public safety personnel and military members (Stelnicki et al., 2021; Ioachim et al., 2024).

The FD/FR framework is thoughtfully embedded into the BOS program, offering proactive coping strategies during transitions between work and personal life. BOS integrates the FD/FR model and Stoic principles, aligning with the original Stoic philosophy. The BOS program employs evidence-based proactive strategies to address adverse outcomes from potentially traumatic events and operational stressors. Emerging evidence suggests BOS improves mental health outcomes in PSP (Stelnicki et al., 2021, Ioachim et al., 2024). A revised version of BOS for military SM was launched in December 2023, with future evaluations to include military SM data.

To integrate the newly developed methods into the BOS program or clinical practice, we recommend incorporating the updated FD/FR model and Stoic exercises into the existing curriculum. This involves creating new training modules focused on practical Stoic principles. Workshops and training sessions for trainers and mental health professionals will ensure they are well-prepared to teach and implement these methods. Pilot programs in select military and PSP units can test the effectiveness and feasibility of the updated methods, with data and feedback refining the approach before broader implementation. Encouraging mental health professionals to integrate these exercises into therapy sessions and equipping clients with practical tools will further enhance resilience and well-being. By following these steps, the newly developed methods can be seamlessly integrated into the BOS program and clinical practice, enhancing the psychological resilience of military SM and PSP.

## 6 Conclusion

The updated FD/FR model, integrating Stoic principles, offers a promising approach to enhancing the mental resilience and well-being of military SM and PSP. By embedding Stoic exercises into existing programs and clinical practice, we provide practical tools to proactively mitigate the negative mental health outcomes associated with operational stress. A key strength of the updated FD/FR model is its alignment with the historical and cultural context of military and public safety professions, which have long valued Stoic philosophy. This alignment can facilitate greater acceptance and motivation among military SM and PSP. Additionally, the proactive nature of the FD/FR model addresses a significant gap in early career mental health training, equipping individuals with skills to manage stress and enhance mental resilience. While the updated FD/FR model provides a framework for assessing controllable variables, the line between controllable and uncontrollable factors can often be blurred in high-stress environments, highlighting the need for continuous refinement based on real-world feedback.

We encourage military and PSP organizations to adopt the updated FD/FR model into existing early career training programs while identifying psychological stressors their personnel may potentially face. Stoic-based education that can train military SM and PSP in active approaches to monitor and intervene on mental wellness, disrupting the potentially deleterious effects of chronic exposure to psychologically traumatic events. Pilot programs and workshops can serve as valuable platforms for testing and refining the model. Initial implementation may face resistance from individuals accustomed to traditional training methods, and the effectiveness of the model may vary across different units and contexts. However, if military SM and PSP can learn proactive mechanisms to attend to mental wellness throughout their careers, this may impact the pervasive psychological difficulties currently identified in these populations.

Future research should focus on further validating and refining the updated FD/FR model. Longitudinal studies assessing the long-term impact of the model on mental health outcomes in military SM and PSP are essential. Exploring the integration of the model with other therapeutic approaches could enhance its effectiveness and applicability. By addressing these areas, we can continue to improve the FD/FR model, ensuring it remains a valuable tool for enhancing the mental resilience and well-being of those who serve in high-risk professions.

## Data Availability

The original contributions presented in the study are included in the article/supplementary material, further inquiries can be directed to the corresponding author.
